# Early recurrence after cryoballoon versus radiofrequency ablation for paroxysmal atrial fibrillation: mechanism and implication in long-term outcome

**DOI:** 10.1186/s12872-022-02816-1

**Published:** 2022-09-07

**Authors:** Yue Wei, Yangyang Bao, Changjian Lin, Yun Xie, Qingzhi Luo, Ning Zhang, Liqun Wu

**Affiliations:** Department of Cardiovascular Medicine, Ruijin Hospital, Shanghai Jiao Tong University School of Medicine, 197 Ruijin No.2 Road, Shanghai, 201204 China

**Keywords:** Atrial fibrillation, Cryoballoon ablation, Radio-frequency ablation, Early recurrence, Inflammation

## Abstract

**Background:**

Early recurrence (ER) after catheter ablation for atrial fibrillation (AF) has been considered as a common phenomenon but its mechanism and implication in long-term outcome has not been fully elucidated. We aimed to clarify the relation between post-ablation inflammation and ER after cryoballoon ablation (CBA) or radio-frequency ablation (RFA) and evaluate the clinical significance of ER.

**Methods:**

A total of 154 patients with paroxysmal AF undergoing ablation were consecutively recruited, including 90 patients undergoing RFA (RF group) and 64 patients undergoing CBA (CB group). Myocardial injury and inflammation biomarkers were analyzed before and 6 h, 24 h and 48 h after ablation. Acute early recurrence (AER), non-acute early recurrence (NAER) and late recurrence (LR) was defined as recurrence of atrial tachyarrhythmia during 0–3, 4–90 days and beyond a 90-day blanking period after ablation.

**Results:**

Cardiac troponin I was significantly higher in CB group while C reactive protein (CRP) and Ratio Neutrophil/Lymphocyte were more elevated in RF group. Higher CRP level after RFA was significantly associated with AER in RF group and lower CRP level after CBA was predictive of AER in CB group. In addition, average cryoablation duration was positively correlated with CRP level after CB group. Cox regression revealed that NAER and left atrial diameter were associated with LR in RF group, while AER and NAER were predictive of LR after CBA.

**Conclusions:**

Post-ablation inflammation was greater in RFA than in CBA. Excessive inflammatory response may be an important factor of AER after RFA. AER after CBA was related with lower inflammation and predictive of LR. Further investigations are still warranted to address on these findings.

**Supplementary Information:**

The online version contains supplementary material available at 10.1186/s12872-022-02816-1.

## Introduction

Catheter ablation to achieve pulmonary vein isolation (PVI) is now considered as the cornerstone of catheter ablation for atrial fibrillation (AF). Radiofrequency ablation (RFA) and cryoballoon ablation (CBA) have emerged as two common methods based on PVI, both of which show comparable long-term efficacy and safety [1].

Regarding short-term outcome, early recurrence (ER) during the postoperative blanking period is common after either CBA or RFA, while the underlying mechanism for ER remains still unclear. Studies have shown that inflammation caused by ablation lesion is closely related to ER [2] and that post-ablation inflammation response differed between RFA or CBA [3]. Additionally, although the significant association between ER and late recurrence (LR) beyond blanking period was recognized, the clinical significance of ER in different stage may be distinct between RFA and CBA, especially very early AF recurrence during blanking period. It was reported that very early recurrence was strongly related with LR after RFA [4, 5], while in another study, very early recurrence after CBA is not associated with LR [6].

The aims of present study were to (1) evaluate post-ablation inflammation after RFA and CBA; (2) investigate the relation between inflammation response and ER; (3) examine the association of ER in different stage and LR.

## Materials and methods

### Patients

One hundred and fifty-four paroxysmal AF patients undergoing catheter ablation were consecutively enrolled in this retrospective study from August 2016 to July 2017. Patients diagnosed with paroxysmal AF and resistant to one or more antiarrhythmic drugs were included and assigned to either RF ablation or CB ablation based on the operators’ preference.

### Preoperative preparation

Before ablation, AADs were discontinued for at least five half‐lives, with the exception of amiodarone, which was discontinued for at least 14 days. The CHA_2_DS_2_-VASc score was calculated for all patients and anticoagulation treatment was given according to the score. Patients were properly anticoagulated for at least 4 weeks before ablation procedure. INR was adjusted between 2.0 and 2.5 before ablation in patients treated with warfarin. Anticoagulation was not interrupted in patients treated with non-vitamin K antagonist oral anticoagulant. All patients underwent transthoracic echocardiography to evaluate cardiac structure and cardiac function and transesophageal echocardiograph to exclude left atrial thrombosis.

### RFA strategy

Patients were under local anesthesia of groin with 2% lidocaine and monitored for their vital signs. A decapolar catheter was placed in the coronary sinus. Left atrium was accessed transseptally from the right femoral vein with a SL1 sheath and a SR0 sheath (Abbott). After atrial transseptum, heparin was perfused intravenously with a bolus and activated clotting time (ACT) was monitored and maintained for more than 300 s. Open irrigation ablation catheter and Lasso catheter were placed in left atrium through SL1 and SR0 sheath. Three-dimensional reconstruction of the left atrium and pulmonary veins was performed by those catheters under the guidance of three-dimensional systems (Carto 3, Biosense Webster). Circumferential pulmonary vein ablation was performed to eliminate pulmonary vein potential (PVP) and to achieve bi-directional block between pulmonary vein and left atrium. If heart rhythm converted to atrial tachycardia or atrial flutter, mapping and ablation of atrial tachycardia or atrial flutter was performed. If heart rhythm converted to AF, complex fractionated atrial electrograms (CFAE) ablation was carried out. If atrial arrhythmia persisted, drug cardioversion or electrical cardioversion was performed to restore sinus rhythm and to verify PVI. Ablation energy: 30–35 W for posterior wall, 35–40 W for anterior wall. Cold saline flow rate is 18–25 ml/min.

### CBA strategy

Patients were under local anesthesia of groin with 2% lidocaine and monitored for their vital signs. A decapolar catheter was placed in the coronary sinus. Left atrium was accessed transseptally from the right femoral vein with a steerable 12F sheath (FlexCath, Cryocath, Medtronic), through which cryoballoon (CB, Arctic Front or Arctic Front Advance, diameter 28 mm or 23 mm) and a circular catheter (Achieve, 15 mm or 20 mm in diameter) were placed in the left atrium within FlexCath sheath. After atrial transseptum, heparin was perfused intravenously with a bolus and ACT was monitored and maintained for more than 300 s. Cryoablation was applied for 180 s for each pulmonary vein after it was completely occluded in order to achieve complete PVI. One bonus cryoablation was delivered after PVI was achieved. TTI was monitored for the decision of the cryoablation if PVP was recorded. The cryoablation was continued under those two conditions: (1) isolation of the PV within 60 s (TTI < 60 s); (2) significant delay of the PVP and isolation of the PV within 90 s (PVP delay and TTI < 90 s). Otherwise, the cryoablation was ceased prematurely. During cryoablation of the right sided pulmonary veins, phrenic movement was monitored by continuous phrenic nerve stimulation. Cryoablation was immediately terminated if diaphragmatic weakness or palsy occurred or nadir balloon temperature was lower than – 55 °C. Complete PVI was considered as bidirectional conduction block between pulmonary vein and left atrium. For AF persisting during ablation, cardioversion was applied if the heart rhythm was still AF when the cryoablation had been finished. In case of typical flutter or focal atrial tachycardia, a cryothermal catheter was permitted to isolate cavo-tricuspid isthmus or eliminate focal atrial tachycardia.

### Blood sampling and analysis

The levels of sensitive cardiac troponin I (cTnI), CRP and neutrophil count with lymphocyte count (for calculation of N/L) were measured at the time of admission and again 6 h, 24 h and 48 h after ablation. CTnI normal upper limit is 0.04 ng/ml. CRP normal upper limit is 4 mg/L. The normal level of neutrophils and lymphocytes is 4.0–9.5 × 10^9/L and 1.1–3.2 × 10^9/L.

### Post-ablation management and follow-up

Continuous ECG monitor was performed during 72 h after ablation. OAC was continued at least 8 weeks after procedure and prescription of AADs was allowed during the blanking period at the discretion of the clinical cardiologist. Patients were monitored by 24 h Holter-ECG during follow-up visits every 3 months in the first year and every 6 months thereafter. Acute early recurrence (AER), Non-Acute early recurrence (NAER) and late recurrence (LR) are defined by documentation of AF, atrial flutter or atrial tachycardia for more than 30 s during 72 h after ablation, between 72 h and 90 days after ablation and beyond a 90-day blanking period.

### Data analysis

Continuous variables with normal distribution were here expressed as mean ± standard deviation, and non-normal variables were reported as medians (interquartile range). These were analyzed using independent samples Student’s t-test, analysis of variance or rank sum test as appropriate. Categorical data are presented as counts or percentages, and compared by the chi-square test. Hazard ratios and CIs for hazard ratios were derived from Cox proportional hazards models. A value of P < 0.05 was considered statistically significant. Software SPSS 16.0 was used for statistical analysis (SPSS Inc., Chicago, IL, US).

## Results

### Baseline characteristics and procedural results

Among 154 AF patients, 90 patients were treated with RFA (RF group) and 64 patients treated with CBA (CB group). No significant difference of baseline characteristics was found between the two groups. The intervention time was significantly longer in RF group than that in CB group, and the fluoroscopy time was similar between two groups. See Table 1.

**Table 1:** Baseline clinical, echocardiographic and procedural characteristics

	RF group (n = 90)	CB group (n = 64)	*p* value
Age, year	62.7 ± 10.0	60.8 ± 8.9	0.23
Female Gender	32%	27%	0.45
Mean BMI, kg/m^2^	27.9 ± 4.2	26.8 ± 3.4	0.07
AF duration, year	5.8 ± 5.0	5.4 ± 4.2	0.82
*Comorbidities*			
Hypertension	44%	36%	0.29
Diabetes mellitus	17%	11%	0.32
Coronary artery disease	9%	9%	0.92
Heart Failure	4%	5%	0.94
Stroke	6%	9%	0.37
*Echocardiographic parameters*			
LA diameter, mm	43.5 ± 5.0	42.5 ± 4.1	0.19
LVEF, %	61.6 ± 9.0	63.8 ± 6.0	0.09
*Procedure details*			
Procedure duration, min	125.0 ± 29.5	110.0 ± 23.7	0.001
Fluoroscopy duration, min	16.7 ± 6.0	16.9 ± 6.2	0.24

*LA* Left atrium; *LVEF* Left ventricular ejection fraction; *ns* No significant difference

During procedure, PVI was achieved in all patients in RF group and CB group. In RF group, two patients (2.2%) presenting typical atrial flutter underwent cavo-tricuspid isthmus linear ablation, two patients (2.2%) underwent roof linear ablation, seven patients (7.8%) presenting atrial tachycardia underwent focal ablation and eight patients (8.9%) underwent CFAE ablation. Electrical cardioversion was performed in eight patients (8.9%).

In CB group, 23 mm CB was additionally applied in 3 patients (4.7%) in case of difficulty in isolating certain PV while no additional touch-up ablation was performed for completing PVI. Two patients (3.1%) presenting typical atrial flutter underwent cavo-tricuspid isthmus linear ablation using cryothermal catheter. Electrical cardioversion was performed in two patients (3.1%).

### Time course of myocardial injury and inflammation biomarkers

**Fig. 1:**
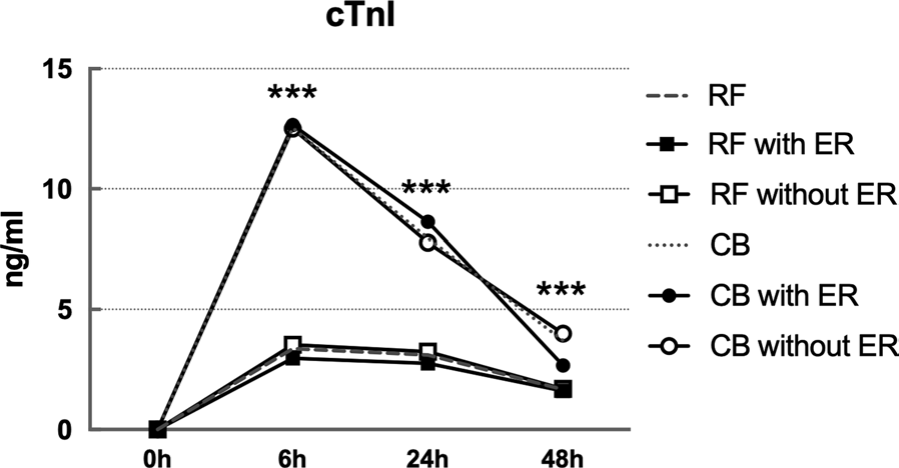
Kinetics of cTnI in CBA and RFA group with and without ER. ***: CB vs. RF, *P*<0.001

**Fig. 2:**
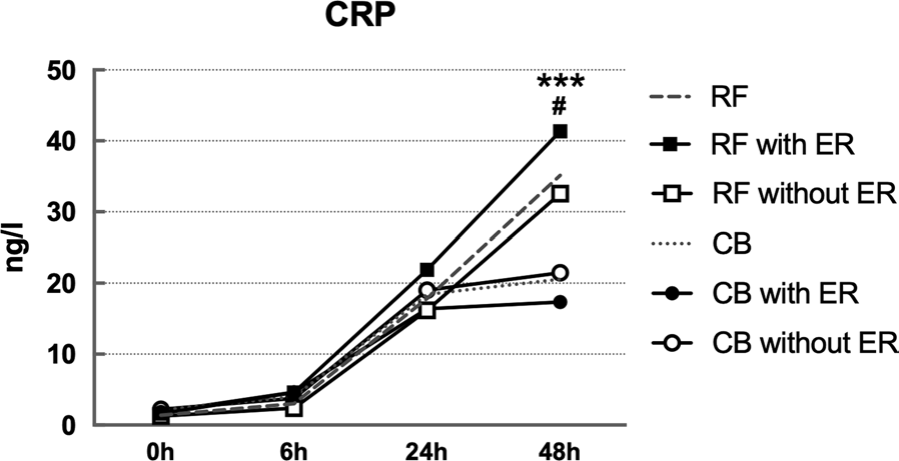
Kinetics of CRP in CBA and RFA group with and without ER. ***: CB vs. RF, P<0.001; #: RF with ER vs RF without ER, *P*<0.05

**Fig. 3:**
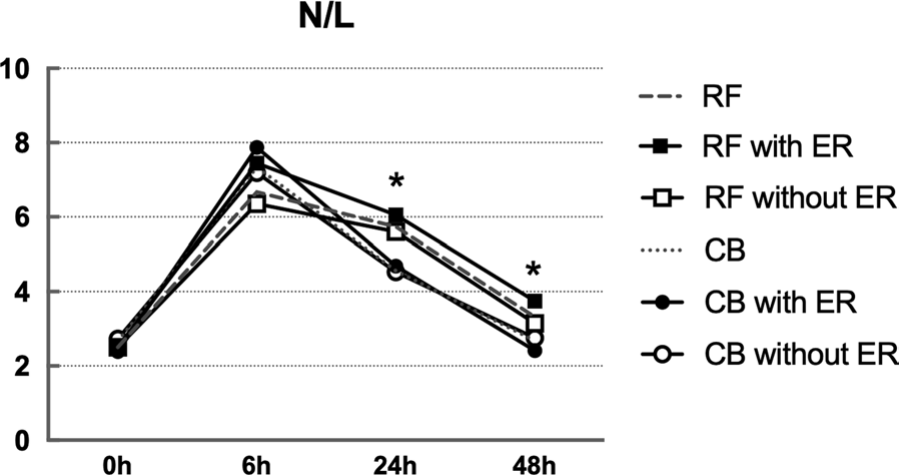
Kinetics of N/L in CBA and RFA group with and without ER. *: CB vs. RF, *P*<0.05

CTnI, CRP and N/L level were elevated in both CB group and RF group after ablation. CTnI peaked at 6 h and level of cTnI after CB ablation was significantly higher than after RF ablation at each time point (*p* < 0.001). CRP reached the peak at 48 h and level of CRP was significantly higher after RF ablation than after CB ablation at 48 h (*p* < 0.001). N/L peaked at 6 h after ablation and N/L was significantly higher after RF ablation than after CB ablation at 24 h (*p* < 0.05). In RF group, higher CRP level was observed in patients experiencing ER than those without ER at 48 h (*p* < 0.05), while similar result was not seen in CB group. There was no difference in cTnI level or N/L ratio between patients with and without ER in either CB group or RF group. See Figs. 1, 2, 3.

**Fig. 4:**
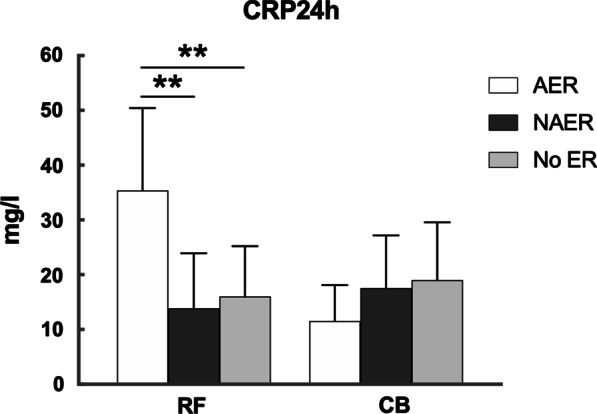
Level of CRP24h in CBA and RFA group with AER, with NAER and without ER. **: RF with AER vs. RF with NAER or RF without ER, *P*<0.01

**Fig. 5:**
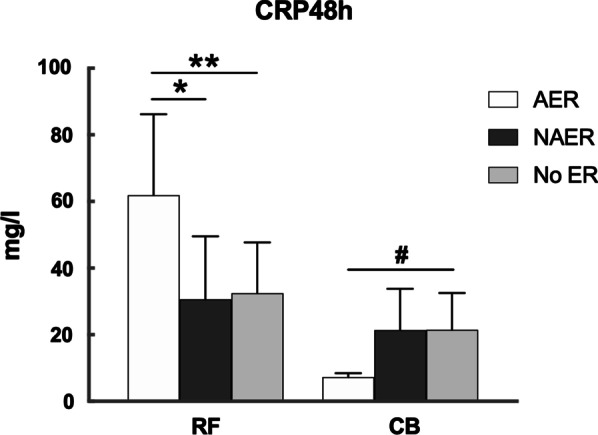
Level of CRP48h in CBA and RFA group with AER, with NAER and without ER. *: RF with AER vs. RF with NAER, *P*<0.05; **: RF with AER vs. RF without ER, *P*<0.01; #: CB with AER vs. CB without ER, *P*<0.05

CRP level after ablation differed among the patients experiencing different stage of ER. In RF group, patients with AER displayed a significantly higher CRP level at 24 h and 48 h compared with patients with NAER and patients without ER. On the contrary, patients with AER in CB group showed a significantly lower CRP level after ablation. See Figs. 4, 5.

### ER after ablation procedure

During a 90-day blanking period, 26 patients in RF group and 14 patients in CB group experienced ER. The incidence of ER was similar between RF group and CB group (28.9% vs. 21.8%, *p* = 0.26). AER and NAER occurred in 11 patients and 17 patients in RF group, and it was observed that 6 patients and 10 patients in CB group experienced AER and NAER. There was no significant difference in the incidence of AER or NAER between the two groups (RF vs. CB, AER: 12.2% vs. 9.4%, *p* = 0.58; NAER: 18.9% vs. 15.6%, *p* = 0.60).

Multivariate logistic regression model including baseline characteristics, myocardial injury and inflammation biomarker revealed that AF duration (OR = 1.220, *p* = 0.004), CRP at 24 h (OR = 1.019, *p* = 0.019) and CRP at 48 h (OR = 1.058, *p* = 0.035) were predictive of AER in RF group and CRP at 48 h (OR = 0.453, *p* = 0.023) was predictive of AER in CB group. Multivariate logistic regression using the same variables did not reveal any significant predictor for NAER in RF group while it was observed that age (OR = 1.170, *p* = 0.027) was associated with NAER in CB group. See Additional file 1: Table S1.

**Fig. 6:**
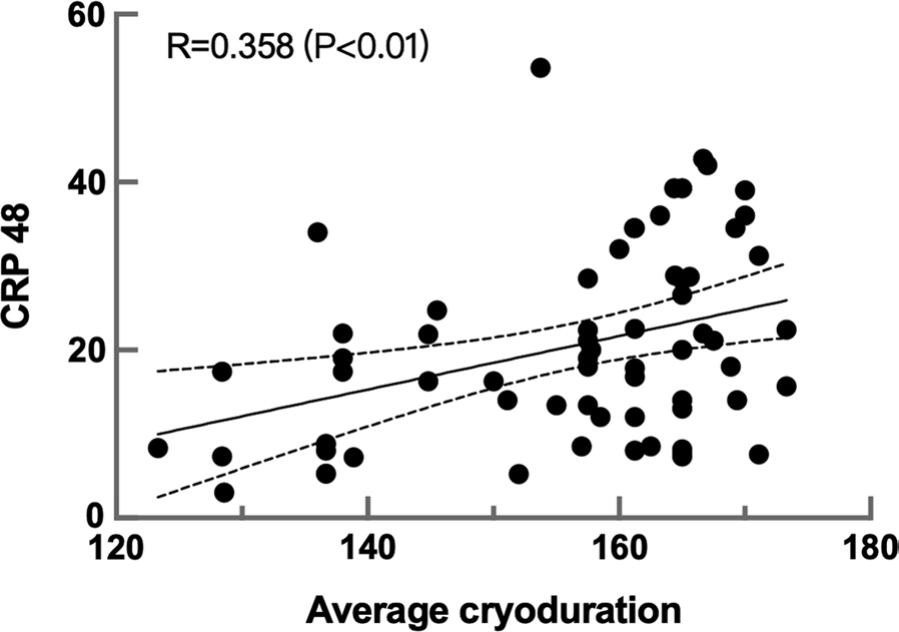
Scatter plot and regression line of CRP48h and average cryoduration

Further investigation for CRP difference in patients with AER after CBA was conducted by including procedural parameter into the analysis. Compared with patients without AER, number of cryoablation application, total cryoablation duration and nadir balloon temperature of each PV were similar in patients with AER. However, a trend of lower average cryoablation duration was noticed in patients with AER than that in patients without AER (see Additional file 1: Table S2). In addition, linear regression analysis revealed a significant correlation between CRP 48 h and average cryoablation duration and the correlation coefficient was 0.358 (*p* < 0.01). The scatter plot and the regression line derived from the regression analysis was shown in Fig. 6.

### Follow-up and LR

**Fig. 7:**
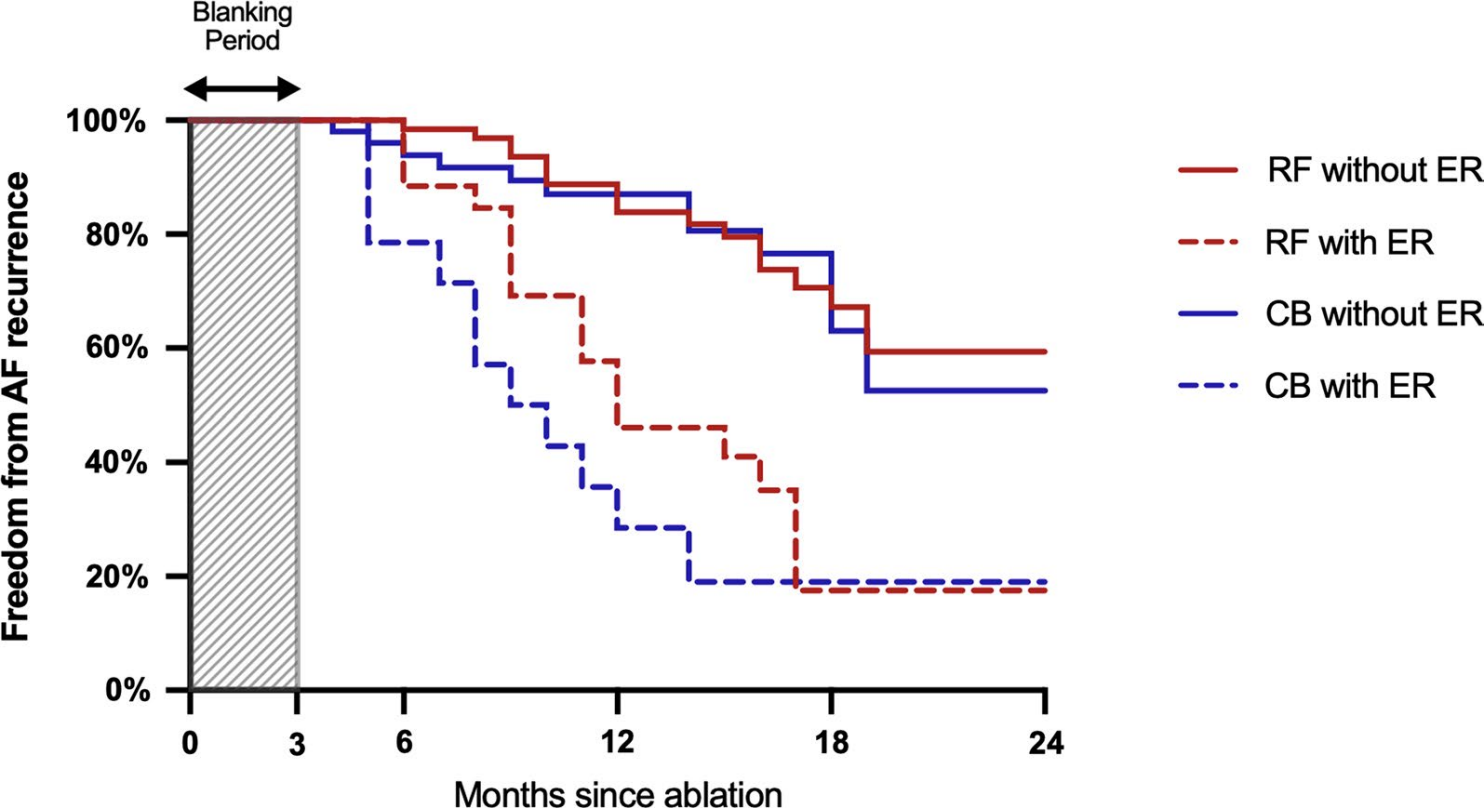
Kaplan–Meier Survival Curve in CBA and RFA group with and without ER. Log-Rank *P*<0.001

After a mean follow-up of 14.1 ± 5.4 months, LR occurred in 36 patients (40.0%) in RF group and 25 patients (39.1%) in CB group. In these patients with LR, 18 patients (50.0%) in RF group and 11 patients (44.0%) in CB group experienced ER. The freedom from AF recurrence in patients with ER was significantly lower than those without ER in both RF group and CB group (RF: *p* < 0.001, CB: *p* < 0.001). Cox regression revealed that LA diameter (HR 1.103, *p* = 0.015) and NAER (HR 4.610, *p* < 0.001) were significantly associated with LR in RF group, and that AER (HR 3.169, *p* = 0.043) and NAER (HR 6.760, *p* < 0.001) was predictive of LR in CB group. See Fig. 7 and Table 2.

**Table 2:** Results of cox regression for LR

	RFA			CBA		
	HR	95%CI	*p* value	HR	95%CI	*p* value
*Univariate analysis*						
Age	0.998	0.965–1.032	0.909	1.045	0.990–1.102	0.108
BMI	0.931	0.853–1.016	0.107	1.224	1.057–1.418	0.007
AF duration	1.055	1.000–1.113	0.052	1.021	0.967–1.078	0.452
Gender, female	0.943	0.464–1.918	0.872	0.680	0.232–1.992	0.482
Hypertension	0.675	0.341–1.333	0.257	1.211	0.544–2.698	0.640
Diabetes	0.868	0.361–2.087	0.751	0.960	0.284–3.240	0.947
Coronary heart disease	2.248	0.984–5.136	0.055	1.330	0.397–4.461	0.644
Heart failure	2.375	0.564–9.992	0.238	2.328	0.544–9.964	0.255
Stroke/TIA	0.381	0.052–2.782	0.341	1.528	0.454–5.142	0.494
LA diameter	1.108	1.025–1.198	0.010	1.011	0.911–1.122	0.835
LVEF	0.991	0.953–1.029	0.631	0.981	0.919–1.046	0.551
CHA_2_DS_2_-VASc score	0.992	0.775–1.270	0.951	1.132	0.770–1.662	0.528
Additional ablation	1.234	0.377–4.034	0.728	–	–	–
CRP 24 h	1.009	0.985–1.033	0.477	0.994	0.952–1.037	0.765
CRP 48 h	1.001	0.984–1.017	0.941	1.019	0.983–1.056	0.303
AER	1.915	0.838–4.376	0.124	2.481	0.846–7.278	0.098
NAER	4.745	2.332–9.655	< 0.001	5.660	2.453–13.059	< 0.001
*Multivariate analysis*						
Age			0.831			0.704
BMI			0.062			0.459
AF duration			0.054			0.330
Coronary heart disease			0.268			0.124
LA diameter	1.103	1.020–1.193	0.015			0.913
AER			0.055	3.169	1.037–9.687	0.043
NAER	4.610	2.243–9.476	< 0.001	6.760	2.845–16.06	< 0.001

## Discussion

The major findings of our study were as follows: (1) systematic inflammation response is greater after RFA than after CBA; (2) excessive post-ablation inflammation is associated with AER in paroxysmal AF patients undergoing RFA but not CBA; (3) NAER strongly predicts LR after either RFA or CBA while AER is associated with LR only after CBA.

### Kinetics of myocardial injury and inflammation biomarkers after CBA and RFA

Our study showed that cTnI was significantly higher after CBA than after RFA, suggesting more important myocardial injury caused by CBA than by RFA. The results of our study are consistent with previous studie [3, 7, 8]. The possible explanation lies in the following two points: First, the ablation range of CBA is larger than that of RFA. Ablation area of CB is half hemisphere, which cause a broader area of ablation lesion in comparison with point-to-point lesion of RFA [9]. Secondly, less influence of blood flow in CBA than RFA. During CBA, blockade of pulmonary vein by CB before cryoablation can cause a fundamental reduction in pulmonary vein blood flow, which can cause more important myocardial injury. Besides ablation, electrical cardioversion was performed in some patients in either group, which could lead to mild rise of myocardial injury biomarker, but the myocardial injury due to cardioversion was lower compared to that induced by ablation.

In our study, RFA showed greater CRP and N/L peak level compared with CBA. The underlying mechanism of this result may be due to lesion formation of different mechanisms: RFA-induced thermal-lesion is characteristic of heterogeneous myocardium coagulation-like necrosis with disorganization of tissue structure; CBA-induced cryo-lesion is typical of myocardial necrosis with preservation of cell ultrastructure with less endothelial damage and thrombosis. The former is more likely to cause intensive and persistent inflammatory response, while the latter results in lower inflammatory response than the former [10, 11]. Even after ablation (6 to 8 weeks after ablation), pathologic examination revealed chronic inflammatory signs in RF-ablated tissues, whereas there was no chronic inflammatory sign in cryoablated tissues, suggesting that RFA-induced tissue lesion is more pro-inflammatory and more likely to cause persistent inflammation reaction [12]. As a result, RFA tends to cause greater inflammation while CBA produces more myocardial damage.

### ER after RFA and associated factors

Mechanism of ER after AF Ablation remains to be explained. In our study, CRP level after RFA was much higher in patients with AER than other patients, suggesting that post-ablation inflammation seems to play a crucial role in early stage of blanking period. This result is consistent with literature. Koyama et al. [13] found that serum CRP level was higher in AF patients undergoing RFA with acute relapse within 3 days than those who had early relapse from day 4 to day 30. Similar result was also observed that CRP level after RFA was associated with recurrence within 3 days but not with recurrence beyond 3 days [14]. Moreover, studies have shown that anti-inflammatory treatment such as glucocorticoid or colchicine can effectively reduce the incidence of AER [13, 15]. To conclude, all above information suggests that AER after RFA is related with activation of inflammation.

The results of our study suggests that NAER, but not AER, was associated with LR after RFA. Since AER is considered to be mediated by excessive inflammatory response, it is reasonable to speculate that a proportion of AER could be transient and have no impact on long-term outcome. Similar result was seen in the Koyama’s study [16] where patients undergoing RFA for PAF with AER showed better successful rate than patients with NAER after a follow-up of 6 months (76% vs. 30%). Besides, Lim et al. [14] found that patients with AER displayed less recurrence rate than patients with recurrence between 30 and 90 days after RFA. However, in our study, AER tended to be related with LR with a marginal p value, and distinct results were reported that ERs in different stages (including AER and NAER) are associated with LR regardless of time from RFA procedure to the first episode of ER [6, 7]. These results suggest that some AERs could be caused by inflammation, while other AERs may have the same pathophysiological mechanisms as NAER, and the latter is more likely to cause LR. Further larger clinical trials are still necessary to identify the clinical implication of ERs in different stages.

### Clinical significance of ER after CBA and associated factors

Little was known about the cause of very early relapse after CBA. In our study, we discovered that CRP level in patients with AER after CBA was lower, which rules out the possibility of AER caused by excessive inflammation. Further investigation revealed that shorter average cryoduration was correlated with lower CRP, which shed light on the possibility that the lower atrial inflammation induced by CBA could be associated with AER. By reviewing the cases, we summarized the reasons for shorter average cryoduration: (1) Difficulty in achieving PVI required more attempts of cryoablation; (2) Shorter cryoduration due to low balloon temperature; (3) Early termination of cyroablation owing to phrenic nerve palsy. Reasons above are likely to cause insufficient ablation of certain PVs and induce lower inflammation.

Cryo-lesion is known to be less pro-inflammatory. Therefore, prevalence of AER after CBA was supposed to be lower than RFA. However, prevalence of AER was actually similar between CBA and RFA in our study. Our study demonstrated that AER could be predicted by lower inflammation and related with reduced average cryoduration. Therefore, we speculate that insufficient cryo-lesion, which could be presented as lower inflammation, might be responsible for PV reconnection or reactivation of pro-arrhythmic matrix during the early phase of post-CBA. By assessment of PET/MRI, Kiuchi found that CBA induced atrial inflammation was strongly associated with lesion maturation, which could be important to the ablation success [17]. Since the inflammation after AF ablation is double-edged, optimal inflammation could consolidate the ablation lesion while lower inflammation may unmask lesion gap and consequently lead to early recurrence. Cryotherapy creates less pro-inflammatory lesion than RF technique. And based on our results, shorter cryoduration induces lower inflammation, while sufficient cryotherapy is of significance for durable PVI. Further studies are still warranted to confirm our findings.

In CBA, both AER and NAER were associated with LR, while risk of LR was higher in patients with NAER. It has been well recognized that blanking period recurrence after CBA is closely related to long-term recurrence, of which the mechanism may be due to excessive inflammation, autonomic nerve change, PVP recovery and reactivation of pro-arrhythmic matrix [18–20]. However, our study has ruled out the possibility of AER caused by excessive inflammation. On the contrary, lower inflammatory responses may represent insufficient cryo-lesion and associated with AER. However, this hypothesis requires further investigation to confirm. The relation between AER and long-term outcome is of great importance in the definition of blanking period and the management of AER patients.

### Research limitations

Our study has the following limitations: (1) Although our study enrolled consecutive patients, it is a retrospective and observational study; (2) This was a single-center study and the patient number was limited; (3) The ablation region was extended in some patients treated with RFA than the patients treated with CBA. In certain patients in RF group, the elimination of atrial tachycardia, atrial flutter and CFAE were performed after PVI; (4) The follow-up was performed using 24 h Holter-ECG and additional ECG in case of symptoms, which may underestimate the recurrence rate; (5) Deficit of procedural data of patients with recurrence undergoing repeat ablation. PV reconnection is considered to be an important factor in either ER or LR, and due to the lack of the data, we were unable to elucidate the role of PV reconnection in the mechanism of AF recurrence in addition to inflammation. Larger prospective clinical studies are warranted to further explore the mechanism of ER in different stage and the clinical significance of AER after CBA.

## Conclusion

Despite CBA exhibiting more pronounced myocardial injury than RFA, systematic inflammation response was more intense after RFA. AER after RFA may be caused by excessive inflammatory response and was not associated with LR. AER after CBA was related with lower inflammation and predictive of LR. Further investigations are warranted to further address on these findings.

## Supplementary Information

**Additional file 1. Table S1.** Results of logistic regression for AER and NAER. **Table S2.** Procedure parameters of patients with AER or without AER in CB group.

## Data Availability

The datasets used and analysed during the current study available from the corresponding author on reasonable request.
