# Supplementary material for: Early recurrence after cryoballoon versus radiofrequency ablation for paroxysmal atrial fibrillation: mechanism and implication in long-term outcome
Source: BMC Cardiovasc Disord. 2022 Sep 7;22:400. doi: 10.1186/s12872-022-02816-1 (PMC9450458; doi:10.1186/s12872-022-02816-1)
Supplement: Supplementary file 1 — Additional file 1. Table S1. Results of logistic regression for AER and NAER. Table S2. Procedure parameters of patients with AER or without AER in CB group. [file 12872_2022_2816_MOESM1_ESM.docx]

**Supplementary Materials**

Supplementary Table 1: Results of logistic regression for AER and NAER.

Supplementary Table 2: Procedure parameters of patients with AER or without AER in CB group.

Supplementary Table 1 Results of logistic regression for AER and NAER

| AER | RF | | |  | CB | | |
| --- | --- | --- | --- | --- | --- | --- | --- |
|  | OR | 95%CI | P value |  | OR | 95%CI | P value |
| Age |  |  | 0.133 |  |  |  | 0.526 |
| BMI |  |  | 0.923 |  |  |  | 0.124 |
| AF duration | 1.063 | 1.064-1.399 | 0.004 |  |  |  | 0.735 |
| Gender, female |  |  | 0.375 |  |  |  | 0.448 |
| Hypertension |  |  | 0.860 |  |  |  | 0.597 |
| Diabetes |  |  | 0.184 |  |  |  | 0.395 |
| Coronary heart disease |  |  | 0.858 |  |  |  | 0.980 |
| Heart failure |  |  | 0.133 |  |  |  | 0.353 |
| Stroke/TIA |  |  | 0.666 |  |  |  | 0.820 |
| LA diameter |  |  | 0.403 |  |  |  | 0.948 |
| LVEF |  |  | 0.183 |  |  |  | 0.884 |
| CHA_2_DS_2_-VASc score |  |  | 0.558 |  |  |  | 0.824 |
| Additional ablation |  |  | 0.980 |  |  |  | - |
| Troponin 24h |  |  | 0.178 |  |  |  | 0.624 |
| CRP 24h | 1.109 | 1.017-1.209 | 0.019 |  |  |  | 0.728 |
| N/L 24h |  |  | 0.307 |  |  |  | 0.545 |
| Troponin 48h |  |  | 0.378 |  |  |  | 0.476 |
| CRP 48h | 1.058 | 1.004-1.115 | 0.035 |  | 0.453 | 0.229-0.899 | 0.023 |
| N/L 48h |  |  | 0.050 |  |  |  | 0.141 |

| NAER | RF | | |  | CB | | |
| --- | --- | --- | --- | --- | --- | --- | --- |
|  | OR | 95%CI | P value |  | OR | 95%CI | P value |
| Age |  |  | 0.249 |  | 1.170 | 1.018-1.343 | 0.027 |
| BMI |  |  | 0.409 |  |  |  | 0.488 |
| AF duration |  |  | 0.949 |  |  |  | 0.859 |
| Gender, female |  |  | 0.840 |  |  |  | 0.256 |
| Hypertension |  |  | 0.374 |  |  |  | 0.537 |
| Diabetes |  |  | 0.923 |  |  |  | 0.644 |
| Coronary heart disease |  |  | 0.656 |  |  |  | 0.271 |
| Heart failure |  |  | 0.108 |  |  |  | 0.082 |
| Stroke/TIA |  |  | 0.263 |  |  |  | 0.590 |
| LA diameter |  |  | 0.341 |  |  |  | 0.576 |
| LVEF |  |  | 0.671 |  |  |  | 0.786 |
| CHA_2_DS_2_-VASc score |  |  | 0.387 |  |  |  | 0.253 |
| Additional ablation |  |  | 0.645 |  |  |  | - |
| Troponin 24h |  |  | 0.760 |  |  |  | 0.300 |
| CRP 24h |  |  | 0.105 |  |  |  | 0.404 |
| N/L 24h |  |  | 0.072 |  |  |  | 0.620 |
| Troponin 48h |  |  | 0.608 |  |  |  | 0.335 |
| CRP 48h |  |  | 0.274 |  |  |  | 0.703 |
| N/L 48h |  |  | 0.156 |  |  |  | 0.639 |

Supplementary Table 2 Procedure parameters of patients with AER or without AER in CB group

|  | AER | No AER | P value |
| --- | --- | --- | --- |
| Total cryoduration (s) | 1352.2±186.6 | 1363.9±135.8 | 0.848 |
| Application number | 9.3±0.8 | 8.7±1.0 | 0.110 |
| Average cryoduration (s) | 145.1±17.3 | 157±11.8 | 0.018 |
| LSPV nadir temperature (℃) | -46.7±2.8 | -47.8±5.6 | 0.621 |
| LIPV nadir temperature (℃) | -43.3±4.8 | -45.3±3.9 | 0.251 |
| RSPV nadir temperature (℃) | -52.2±2.5 | -51.2±5.1 | 0.654 |
| RIPV nadir temperature (℃) | -44.5±2.4 | -47.7±5.5 | 0.176 |
